# Computed tomography-guided cutting needle biopsy for lung nodules: when the biopsy-based benign results are real benign

**DOI:** 10.1186/s12957-022-02647-6

**Published:** 2022-06-04

**Authors:** Hui Hui, Gao-Lei Ma, Hai-Tao Yin, Yun Zhou, Xiao-Mei Xie, Yong-Guang Gao

**Affiliations:** 1 Department of Radiation Oncology, Xuzhou Central Hospital, 199 Jiefang Road, Jiangsu Xuzhou, China; 2 Department of Radiation Treatment, Xuzhou First People’s Hospital, 269 Daxue Road, Xuzhou, Jiangsu China; 3 Radiology Department, Xuzhou Central Hospital, Xuzhou, China

**Keywords:** Computed tomography, Cutting needle biopsy, Lung nodule, Benign

## Abstract

**Background:**

Computed tomography (CT)-guided cutting needle biopsy (CNB) is an effective diagnostic method for lung nodules (LNs). The false-negative rate of CT-guided lung biopsy is reported to be up to 16%. This study aimed to determine the predictors of true-negative results in LNs with CNB-based benign results.

**Methods:**

From January 2011 to December 2015, 96 patients with CNB-based nonspecific benign results were included in this study as the training group to detect predictors of true-negative results. From January 2016 to December 2018, an additional 57 patients were included as a validation group to test the reliability of the predictors.

**Results:**

In the training group, a total of 96 patients underwent CT-guided CNB for 96 LNs. The CNB-based results were true negatives for 82 LNs and false negatives for 14 LNs. The negative predictive value of the CNB-based benign results was 85.4% (82/96). Univariate and multivariate logistic regression analyses revealed that CNB-based granulomatous inflammation (*P* = 0.013, hazard ratio = 0.110, 95% confidential interval = 0.019–0.625) was the independent predictor of true-negative results. The area under the receiver operator characteristic (ROC) curve was 0.697 (*P* = 0.019). In the validation group, biopsy results for 47 patients were true negative, and 10 were false negative. When the predictor was used on the validation group, the area under the ROC curve was 0.759 (*P* = 0.011).

**Conclusions:**

Most of the CNB-based benign results were true negatives, and CNB-based granulomatous inflammation could be considered a predictor of true-negative results.

## Introduction

Computed tomography (CT)-guided cutting needle biopsy (CNB) is an effective diagnostic method for lung masses or nodules due to its mini-invasive nature and high diagnostic accuracy [1–8]. CNB-based malignant results can be considered the final diagnosis as the rate of false positives is extremely low (0–0.2%) [9]. A CNB-based-specific benign diagnosis (e.g., tuberculosis, fungal infection, or benign tumors) can also be accepted as the final diagnosis [1–8], enabling patients with suspicious lung lesions to avoid unnecessary surgery.

However, the management of a CNB-based nonspecific benign diagnosis (e.g., chronic inflammation) is challenging because this diagnosis cannot be considered the definite final diagnosis [1–8]. The false-negative rate of CT-guided lung biopsy was reported to be up to 16% [9]. At present, some studies have established some predictors of true- or false-negative findings from CNB-based nonspecific benign results [9, 10]. However, there is a lack of studies which investigated true-negative findings in lung nodules (LNs) with CNB-based nonspecific benign results.

In this study, we determined the predictors of true negatives in LNs with CNB-based benign results.

## Methods

This retrospective study was approved by our ethics committee. The requirement of informed consent was waived due to the retrospective nature.

### Patients

**Fig. 1:**
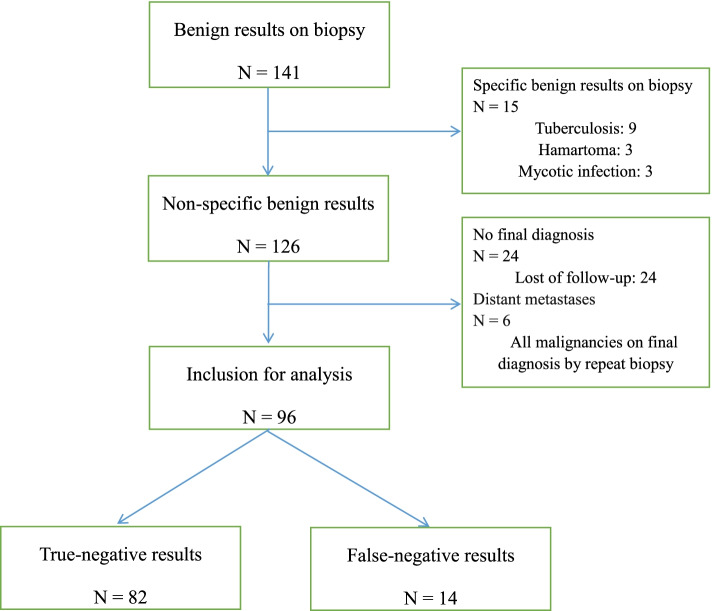
A flowchart of training group in this study

From January 2011 to December 2015, a total of 141 patients with CNB-based benign results from LNs were collected. Among them, 96 patients with CNB-based nonspecific benign results were included in this study as the training group that detected the predictors of true-negative results (Fig. 1). From January 2016 to December 2018, additional 57 patients were included as a validation group that tested the reliability of the predictors.

The decision for CNB was made based on the recommendation of the management of LNs [11]. The inclusion criteria are as follows: (a) patients with LN and (b) patients with CNB-based benign results. The exclusion criteria are as follows: (a) CNB-based-specific benign results, (b) patients with distant metastasis, and (c) lesions without a definite final diagnosis.

### CT-guided CNB procedure

All procedures were conducted by a chest radiologist who had more than 5 years of experience in CT-guided intervention.

Under the guidance of a 16-detector CT (Philips, Cleveland, Ohio, USA), the needle pathway was selected based on the location of LN. The voltage and current were set as 120 kV and 150 mA/s, respectively.

First, an 18G semiautomatic cutting needle (Wego, Weihai, China) was inserted into the lung parenchyma according to the direction of the needle pathway. Then, the repeat CT scan was performed to confirm the location of the needle tip. The needle tip was adjusted appropriately according to the CT result. When the needle tip touched the LN, a specimen was obtained from the LN. When the total samples’ length reached 5–10 mm, the quantity of samples was considered enough. The obtained samples were preserved in 10% formaldehyde for pathological diagnosis. Finally, the CT scan was performed again to check the potential CNB-related complications.

### Definitions

LN is defined as a round or oval lesion ≤ 3 cm that is completely surrounded by pulmonary parenchyma without other abnormalities [1–4]. CNB-based benign results can be divided into specific and nonspecific benign results [10]. Specific benign results include benign tumors and positive microbiologic culture compatible with clinical-radiologic findings. Nonspecific benign results are defined as the CNB-based benign pathological diagnoses such as chronic inflammation or fibrosis that is not specific enough to make a diagnosis.

CNB-based benign results were considered to be true negatives if the lesions were benign upon final diagnosis. A final benign diagnosis could be made in one of the three ways: (a) surgical resection, (b) determination of a specific benign lesion upon pathological analysis of the lung biopsy sample, or (c) a decrease > 20% in lesion diameter, stability in size (without anticancer treatment) over a minimum of 2 years [10–12]. The lesion size was measured on CT by radiologists themselves. If lesions did not meet the criteria mentioned above, final diagnoses were listed as nondiagnostic lesions.

### Statistical analyses

The statistical analyses are calculated by SPSS 16.0 (SPSS Inc., Chicago, IL, USA). Continuous variables are presented as the mean ± standard deviation. Numeric data are calculated by *χ*^2^ tests or Fisher exact probability tests. Predictors of true-negative findings are identified by univariate and multivariate logistic regression analyses. The variables can be included into the multivariate model when the variables are presented as *P* < 0.1 in the univariate analysis. Receiver operator characteristic (ROC) curve is created, and area under the curve is calculated. The differences are considered statistically significant when the *P*-value < 0.05.

## Results

### Training group

In the training group, a total of 96 patients underwent CT-guided CNB for 96 LNs. All of the 96 LNs were diagnosed as CNB-based nonspecific benign. The CNB-based results were true negatives for 82 LNs and false negatives for 14 LNs (Table 1). The negative predictive value (NPV) of the CNB-based benign results was 85.4% (82/96).

**Table 1:** Comparison of baseline data between true and false negatives in training group

	True negative	False negative	*p*-value
Patients number	82	14	
Age (year)	56.3 ± 12.8	64.7 ± 5.3	< 0.001
Sex (male/female)	47/35	7/7	0.610
Smoker	34	5	0.686
Imaging feature			
Size (mm)	18.5 ± 5.8	18.6 ± 5.2	0.989
Solid/sub-solid	81/1	13/1	0.272
Spiculation	38	9	0.214
Pleural retraction sign	32	7	0.440
Cavity	7	1	1.000
Calcification	15	0	0.179
Enlarged hilar or mediastinal lymph nodule (≥ 10 mm)	16	5	0.315
Emphysema	17	3	1.000
Uptake in PET-CT (SUVmax ≥ 2.5)	18 (*n* = 35)^a^	3 (*n* = 8)^a^	0.750
Nodule location			
Right lung/left lung	41/41	7/7	1.000
Upper lobe/non-upper lobe	39/43	6/8	0.744
Details of biopsy procedure			
Lesion — pleura distance (mm)	15.5 ± 14.6	19.5 ± 14.5	0.340
Needle — pleura angle (degrees)	69.0 ± 19.3	64.4 ± 24.5	0.420
Number of specimen	1.6 ± 0.7	1.3 ± 0.5	0.154
Pneumothorax	12	3	0.803
Hemoptysis	16	5	0.315
Tumor marker			
Abnormal CEA (normal: 0–5 ug/L)	6	5	0.009
Abnormal Cyfra21-1 (normal: 0–3.3 ng/ml)	7	2	0.852
Abnormal SCC (normal: 0–2.5 ug/L)	1	3	0.009
Abnormal NSE (normal: 0–16.3 ng/ml)	2	0	1.000
Pathological feature of biopsy			
Granulomatous inflammation	45	1	0.001
Chronic inflammation with alveolar epithelial hyperplasia	14	3	0.987

*CEA* carcinoembryonic antigen, *CT* computed tomography, *NSE* neuron-specific enolase, *PET* positron emission tomography, *SCC* squamous cell carcinoma antigen, *SUV* standardized uptake value

^a^Thirty-five and 8 patients underwent PET-CT examination in true- and false-negative groups, respectively

### Complications

Pneumothorax and hemoptysis were found in 15 (15.6%) and 21 (22.9%) patients, respectively. Only five patients with pneumothorax were treated with chest tube insertion, and the rest of the patients were treated with anti-inflammatory and hemostasis.

### True-negative LNs

**Fig. 2:**
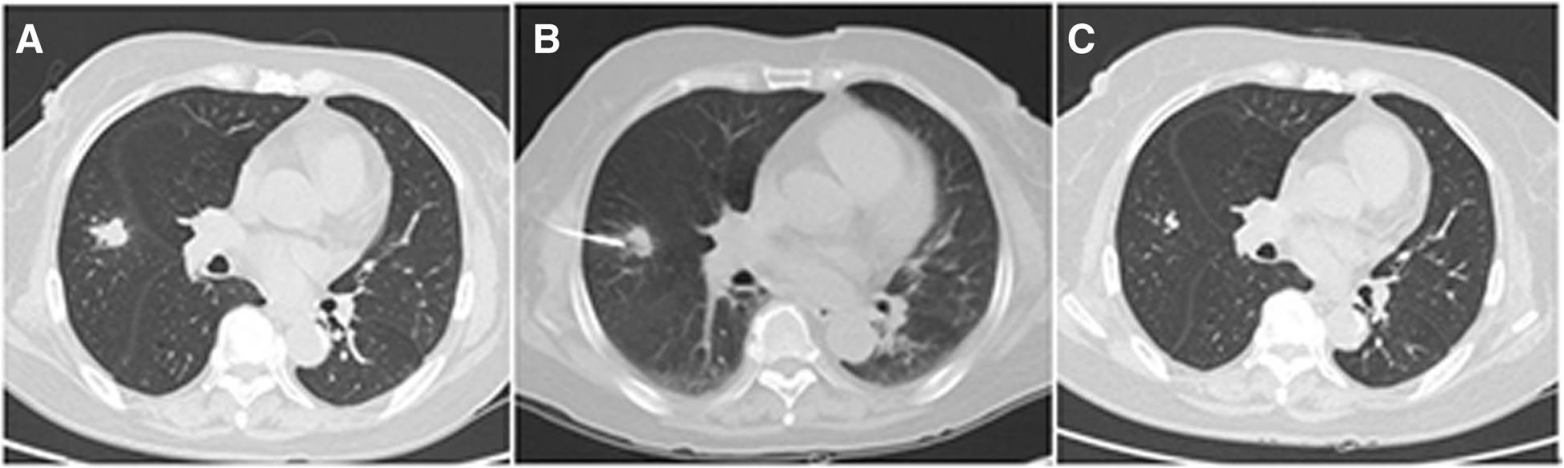
A LN which was presented with CNB-based granulomatous inflammation. **A** A LN located in right middle lobe. **B** The procedure of the lung biopsy. **C** The lesion was significantly resolved after 10 months

Among the 82 true-negative LNs, 56 cases were confirmed by CT follow-up (Fig. 2), and 26 cases were confirmed by surgery. The 26 surgical diagnoses included inflammatory pseudo-tumors (*n* = 20), hamartoma (*n* = 3), granulomatous inflammation (*n* = 2), and bronchial cyst (*n* = 1).

### False-negative LNs

Among the 14 false-negative LNs, 9 cases were confirmed by re-biopsy, and 5 cases were confirmed by surgery. The final diagnoses of the 14 LNs included adenocarcinoma (*n* = 13) and squamous cells carcinoma (*n* = 1).

### Predictor of true negative

Univariate logistic analysis revealed that the predictors of true-negative results included younger age [*P* = 0.024, hazard ratio (HR) = 1.082, 95% confidence interval (CI) = 1.010–1.158], normal carcinoembryonic antigen (CEA) levels (*P* = 0.005, HR = 7.037, 95% *CI* = 1.782–27.784), normal squamous cell carcinoma antigen (SCC) levels (*P* = 0.01, *HR* = 22.091, 95% *CI* = 2.109–231.443), and CNB-based granulomatous inflammation (*P* = 0.015, *HR* = 0.144, 95% *CI* = 0.030–0.684). Multivariate analysis revealed that the independent predictor of true-negative results was CNB-based granulomatous inflammation (*P* = 0.013, *HR* = 0.110, 95% *CI* = 0.019–0.625, Table 2).

**Table 2:** Predictors of true negatives

Variables	Univariate analysis			Multivariate analysis		
	Hazard ratio	95% *CI*	*p*-value	Hazard ratio	95% *CI*	*p*-value
Age	1.082	1.010–1.158	0.024	1.078	0.995–1.169	0.066
Abnormal CEA	7.037	1.782–27.784	0.005	4.228	0.697–25.635	0.117
Abnormal SCC	22.091	2.109–231.443	0.01	13.060	0.801–212.880	0.071
Granulomatous inflammation	0.144	0.030–0.684	0.015	0.110	0.019–0.625	*0.013*

*CI* confident interval, *CEA* carcinoembryonic antigen, *SCC* squamous cell carcinoma antigen

**Fig. 3:**
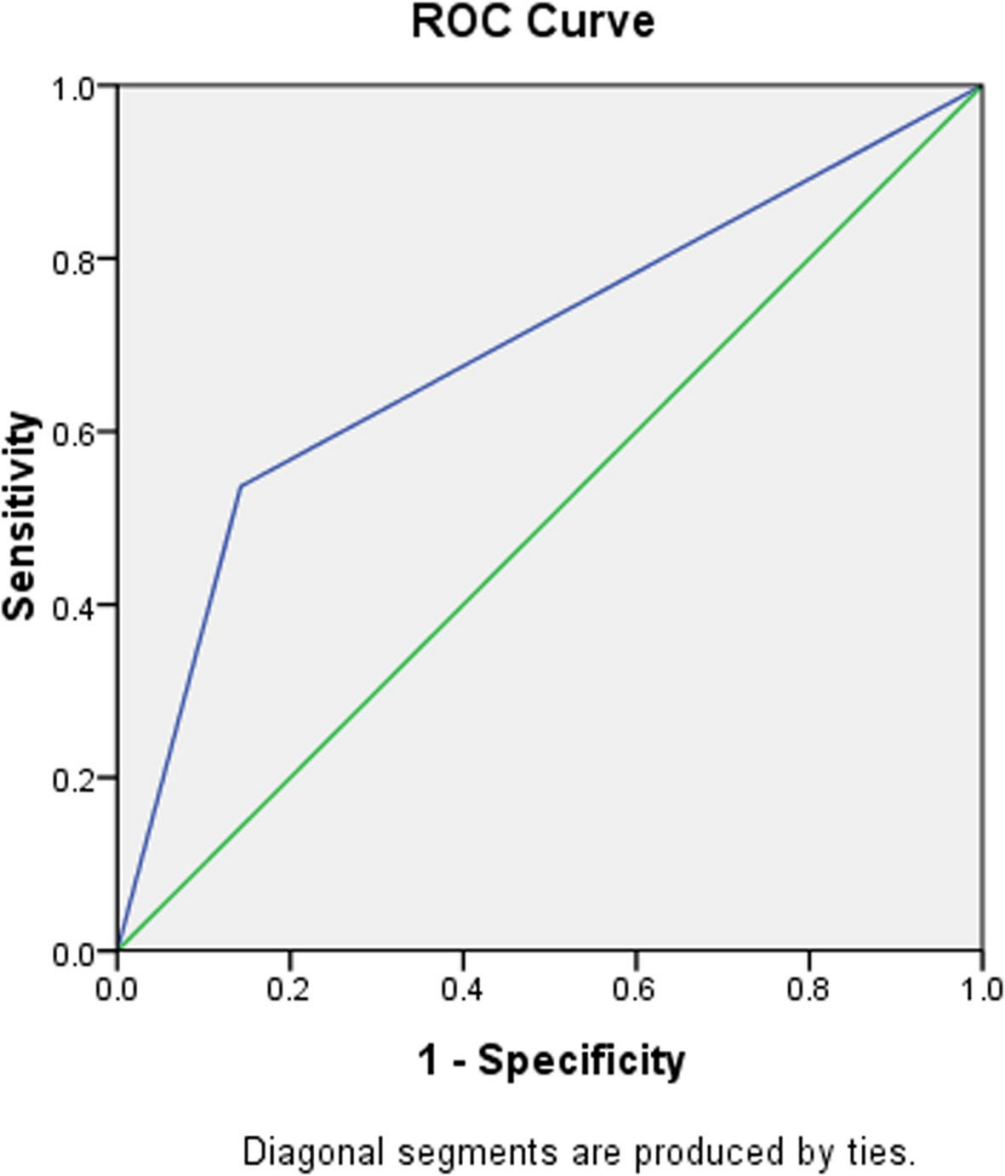
The ROC curve generated using the predictor from training group

A ROC curve was established to test the predictive ability of CNB-based granulomatous inflammation. The area under the ROC curve was 0.697 (*P* = 0.019, Fig. 3).

### Validation group

**Fig. 4:**
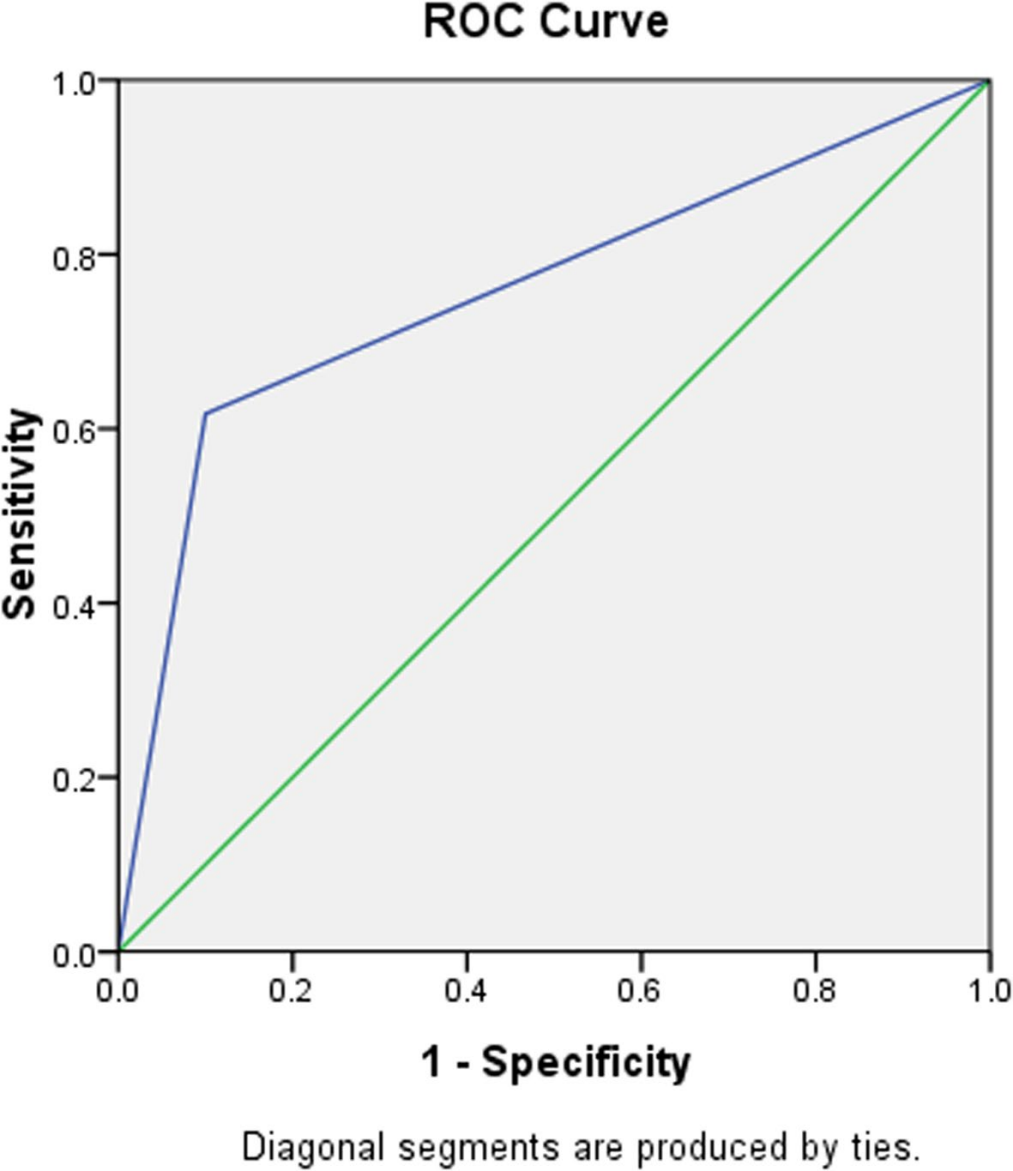
The ROC curve generated using the predictor from validation group

Clinical data of the patients in the validation group (*n* = 57) were used to test the predictive ability of the predictor. There are no significant differences in baseline data between the training and validation groups (Table 3). The biopsy results for 47 patients were true negative, and 10 were false negative. CNB-based granulomatous inflammation was tested by the validation group, and the area under the ROC curve was 0.759 (*P* = 0.011, Fig. 4).

**Table 3:** Comparison of baseline data between training and validation group

	Training group	Validation group	*p*-value
Patients number	96	57	
Age (year)	57.5 ± 12.3	58.2 ± 10.6	0.750
Sex (male/female)	54/42	33/24	0.843
Smoker	39	29	0.217
Imaging feature			
Size (mm)	18.6 ± 5.7	17.8 ± 6.0	0.434
Solid/sub-solid	94/2	55/2	0.992
Spiculation	47	29	0.818
Pleural retraction sign	39	20	0.496
Cavity	8	7	0.427
Calcification	15	3	0.054
Enlarged hilar or mediastinal lymph nodule (≥ 10 mm)	21	9	0.359
Emphysema	20	14	0.592
Nodule location			
Right lung/left lung	48/48	32/25	0.462
Upper lobe/non-upper lobe	45/51	28/29	0.788
Details of biopsy procedure			
Lesion — pleura distance (mm)	16.1 ± 14.6	17.5 ± 13.8	0.558
Needle — pleura angle (degrees)	68.3 ± 20.1	67.2 ± 19.4	0.733
Number of specimen	1.5 ± 0.7	1.4 ± 0.5	0.381
Pneumothorax	15	8	0.790
Hemoptysis	21	14	0.702
Tumor marker			
Abnormal CEA (normal: 0–5 ug/L)	11	7	0.879
Abnormal Cyfra21-1 (normal: 0–3.3 ng/ml)	9	7	0.570
Abnormal SCC (normal: 0–2.5 ug/L)	4	2	1.000
Abnormal NSE (normal: 0–16.3 ng/ml)	2	2	0.992
Pathological feature of biopsy			
Granulomatous inflammation	46	30	0.948
Chronic inflammation with alveolar epithelial hyperplasia	17	13	0.442
True negative/false negative	82/14	47/10	0.626

*CEA* carcinoembryonic antigen, *SCC* squamous cell carcinoma antigen, *NSE* neuron-specific enolase

## Discussion

This study determined the predictors of true-negative results for LNs with CNB-based benign results. First of all, the NPV in the training group of this study was 85.4%, which is within the range demonstrated by previous studies regarding CT-guided CNB for lung lesions (78−90%) [9, 10, 12]. This NPV value may indicate that most CNB-guided benign results are reliable. Among the 82 cases with true-negative results, 56 cases (68.2%) were confirmed by CT follow-up. This result indicates that CT follow-up is a reasonable management for LNs with CNB-based benign results.

Our study found that CNB-based granulomatous inflammation was an independent predictor of true-negative results. The true-negative group had a significantly higher rate of cases with CNB-based granulomatous inflammation than the false-negative group (54.9% vs. 7.1%, respectively; *P* = 0.001). A multicenter study of the malignant risk of CNB-based nondiagnostic results revealed that granulomatous inflammation, abscess, and organizing pneumonia were predictors of true-negative results [13]. Similarly, Liu et al. [12] have found that CNB-based chronic inflammation with fibroplasias is a predictor of true-negative results. Granulomatous inflammation is considered an important step to the formation of organizing pneumonia or inflammatory pseudotumors [12, 14, 15]. Therefore, it is reasonable that CNB-based granulomatous inflammation may indicate a true-negative result.

Granulomas are organized inflammatory infiltrates characterized by a core of macrophages, epithelioid, and multinucleated giant cells and a corona of lymphocytes and a few to many fibroblasts [16]. A worldwide study showed that infection and sarcoidosis were the most common causes of granulomatous inflammation [16]. Granulomatous inflammation is one of the most common sources of false positives in lung biopsy rather than false negatives [17]. Therefore, a CNB-based granulomatous inflammation result may assure the patient and physician that it is indicative of a benign process [10].

Abnormal tumor marker levels were not found to be associated with false-negative results, although univariate logistic analysis demonstrated that abnormal CEA and SCC levels were predictors of false-negative results. Multivariate logistic regression analysis showed that abnormal SCC had a strong tendentiousness for indicating false negative, however without significance (*P* = 0.071). This result may be attributed to the limited sample size. Also, previous studies did not find any tumor marker level to be associated with false negatives from CNB-based benign results [9, 10, 12]. Further studies with larger sample size are needed.

Kim et al. [10] found that partial-solid lesions were a risk factor for false-negative CNB-based benign results (*HR* = 3.95, *P* = 0.022). Many studies suggest that regular CT follow-up is the predominant management of partial-solid LNs [18, 19]. Therefore, only two cases with partial-solid LNs were included in the training group.

A ROC curve was generated to test the predictive ability of CNB-based granulomatous inflammation, with an area under the ROC curve of 0.697 (*P* = 0.019). An additional validation group with 57 cases tested this predictive factor again and demonstrated that the area under the ROC curve was 0.759 (*P* = 0.011). These results may indicate an impressive predictive ability of CNB-based granulomatous inflammation.

There were some previous studies which used PET-CT or tumor markers to distinguish malignant and benign LNs [20, 21]. The previous results also indicated that PET-CT and serum CEA/Cyfra21-1 had good ability of differential diagnosis of LNs with the area under the ROC curve of 0.678–0.863 [20, 21]. Compared to those previous studies, this study had some differences. First, the previous studies included all LNs, while our study only collected the LNs with the initial CNB-based benign results. Second, previous studies mainly assessed the radiological and clinical features between malignant and benign LNs. Our study found the CNB-based benign pathological feature could help to distinguish true- and false-negative results.

The present study had some limitations. First, the major limitation of this study was the retrospective design, which led to selection bias. Second, there is no standard criterion for the quantity of CNB samples that need to be collected. Instead, we collected the samples in accordance with our experience. Although the sample number was not associated with true-negative results, it may have otherwise biased our findings. Third, the PET-CT data were incomplete.

## Conclusion

In conclusion, although further clinical researches are needed, our study indicated that most of the CNB-based benign results were true negatives. CNB-based granulomatous inflammation could be considered as the predictor of true-negative results.

## Data Availability

The data that support the findings of this study are available from the corresponding author upon reasonable request.

## Abbreviations

CNB: Cutting needle biopsy
CT: Computed tomography
LN: Lung nodule
NPV: Negative predictive value
PET-CT: Positron emission tomography-CT
